# Multiple Thoracic Wall Blocks for Awake Breast Surgery: A Case Report

**DOI:** 10.4274/TJAR.2023.231472

**Published:** 2023-12-27

**Authors:** Yavuz Gürkan, İlayda Kalyoncu, Doğa Şimşek, Mete Manici

**Affiliations:** 1 Koç University Faculty of Medicine, Koç University Hospital, Department of Anaesthesiology and Reanimation, İstanbul, Turkey

**Keywords:** Awake surgery, breast surgery, PECS block, serratus anterior plane block, thoracic paravertebral block

## Abstract

Awake breast surgeries under nerve blocks have been a challenge for anaesthesiologists, and different block combinations have been used for surgery under sedation. Thoracic paravertebral block (TPVB) was thought to be sufficient alone for surgical anaesthesia of the breast. We performed a combination of TPVB, pectoralis nerve I block, and serratus anterior plane block for awake breast surgery in an elderly patient with serious comorbidities. Surgical anaesthesia was achieved, excluding skin incision. Any regional anaesthesia technique alone is not sufficient; rather, multiple thoracic wall blocks are needed for surgical anaesthesia of the breast.

**Main Points**

• It was thought that thoracic paravertebral block (TPVB) alone was sufficient for surgical anaesthesia of the breast.

• We performed a combination of TPVB, pectoralis nerve I block and serratus anterior plane block for segmental mastectomy.

• Surgical anaesthesia was achieved excluding skin incision.

• Multiple thoracic wall blocks are needed for surgical anaesthesia of the breast.

## Introduction

Oncologic breast surgeries are mostly performed under general anaesthesia. However, elderly patients with serious comorbidities may not be well suited for general anaesthesia. Awake breast surgeries under nerve blocks have been a challenge for anaesthesiologists, and different block combinations have been used for surgery under sedation.^1^ Among them, thoracic paravertebral block (TPVB) has been the most studied.^2^ In this case report, after obtaining written informed consent for publication, we would like to share our experience regarding awake breast surgery under nerve blocks. This manuscript adhere to the case reports [CARE guidelines (for CAse REports)] statement.

## Case Description

An 85-year-old female (height 150 cm, weight 72 kg, American Society of Anesthesiologists Physical Status III) with a history of hypertension, atrial fibrillation, congestive heart failure, coronary artery disease, and pulmonary hypertension was scheduled for segmental mastectomy due to a mass in the upper outer quadrant of the left breast. Segmental mastectomy was planned under regional anaesthesia using ultrasound-guided TPVB with serratus anterior plane and pectoral nerve blocks. Following premedication with 1 mg of intravenous midazolam, standard monitors (SpO_2_, electrocardiography, non-invasive blood pressure) were applied, and the patient received 4 L of oxygen per minute via a face mask. TPVB was performed in the prone position in the operating room. Before block performance, 25 µg of intravenous (IV) fentanyl was administered. After skin preparation with 10% povidone-iodine, a linear ultrasound probe of GE Logiq P9 (Gyeonggi-do, Republic of Korea) was placed parallel to the vertebral spine at the T4 level and shifted 2-3 cm laterally to obtain the appropriate visualization. Following the identification of the pleura, transverse process, and paravertebral space, a 22 G 50 mm needle (BBraun ultra-360, Melsungen, Germany) was inserted caudal to the cranial direction using an in-plane approach. After confirming the position of the needle tip and observing tenting of the pleura with 1 mL of local anaesthetic (LA), 20 mL of LA mixture consisting of 7 mL of 0.5% bupivacaine, 5 mL of 2% lidocaine, 8 mL of isotonic sodium chloride was administered for the blocks at the T2 and T4 levels.

In the supine position, pectoralis nerve block was performed using 10 mL of the same LA mixture. The probe was placed medially to the coracoid process in the transverse position underneath the clavicle. The third rib, thoracoacromial artery, and pectoralis major and minor muscles were identified. The needle was inserted using an in-plane approach, and LA mixture was administered into the fascia between the pectoralis major and minor muscles.

A serratus anterior plane block was performed at the level of the fifth rib at the mid-axillary level. The probe was moved inferiorly down to the fifth rib. The serratus anterior muscles were visualized. Finally, the needle was inserted using an in-plane approach and advanced caudal to cranial direction until the needle tip was beneath the serratus muscles. Subsequently, 10 mL of the same LA mixture was injected under the fascia of the serratus muscles. Surgery started 30 min after block performance. Fentanyl 50 µg and propofol 10 mg IV were administered immediately before surgery. The patient felt pain only during the skin incision. Wound infiltration with 10 mL of 0.25% prilocaine was provided by the surgical team. Supplementary doses of propofol (total 70 mg) were administered to achieve sedation during surgery. At the end of surgery, paracetamol (1 g IV) was administered for postoperative analgesia. Surgery lasting 55 min was completed uneventfully. On follow-up, the patient reported a pain score of 4 on the numeric rating scale 1 h after surgery. Tramadol 50 mg IV was administered. The patient was discharged the next day without complications and was completely satisfied with the course of treatment.

## Discussion

The risks of general anaesthesia in elderly patients with serious comorbidities are sufficiently high to conclude that there is a need for alternative techniques for surgical anaesthesia. To provide surgical anaesthesia for breast surgery, the clinician must first consider the breast and the superficial tissue innervated by the cutaneous branches of the intercostal nerves through T2-T6 levels. Second, deep layers such as the pectoralis major muscle and its fascia innervated by lateral and medial pectoral nerves, serratus anterior muscle, and latissimus dorsi muscle innervated by long thoracic nerve and thoracodorsal nerve should be taken into account.^2,3^ Our combination of TPVB at two levels (T2-T4), PECS I, and serratus anterior plane blocks should have been adequate for surgical anaesthesia in segmental mastectomy. Although the patient did not feel any pain during deeper dissections, infiltration anaesthesia with 10 mL of 0.25% prilocaine was required for the skin incision. This may be due to the relatively dilute LA concentration we administered. The purpose of our dilute LA choice was to avoid potential LA toxicity due to multiple nerve blocks and infiltration anaesthesia.

It was thought that TPVB would be sufficient for surgical anaesthesia of breast surgery if adequate sedation was provided.^4^ Pangthipampai et al.^5^ reported that even multiple-level TPVB was not adequate to provide surgical anaesthesia. Unfortunately, the depth of sedation is highly variable, ranging from inadequate sedation to almost deep enough to resemble general anaesthesia without securing the airway with a laryngeal mask airway or tracheal intubation. Some authors still suggest that multiple-level TPVB block is all that is needed for most breast surgeries.^6^ In our case, we observed that even in segmental mastectomy there is a need for additional interventions. It is essential to closely monitor the pain and sedation level of patients by both the anaesthesiologist and surgeon throughout the procedure.

## Conclusion

Multiple thoracic wall blocks are required for surgical anaesthesia of the breast. Any regional anaesthesia  technique is not sufficient when used alone for major breast surgery that involves dissection of the pectoralis major muscle and its fascia and possibly also the serratus anterior and latissimus dorsi muscles. Even when using multiple blocks covering all known nerves of the breast, infiltration anaesthesia by the surgeon might be required for skin incision. The search for the ideal block or block combinations for surgical anaesthesia of the breast continues.
